# Supplementary material for: Ultrafast magnetization enhancement via the dynamic spin-filter effect of type-II Weyl nodes in a kagome ferromagnet
Source: Nat Commun. 2024 Mar 18;15:2410. doi: 10.1038/s41467-024-46604-1 (PMC10948858; doi:10.1038/s41467-024-46604-1)
Supplement: Supplementary file 1 — Supplementary Information [file 41467_2024_46604_MOESM1_ESM.pdf]

Supplementary Information of "Ultrafast magnetization enhancement via the dynamic spin-filter effect of type-II Weyl nodes in a kagome ferromagnet"

Xianyang Lu,[1, 2, 3 #] Zhiyong Lin,[4, 5, #] Hanqi Pi,[6, 7, 8, #] Tan Zhang,[9] Guanqi Li,[10] Yuting Gong,[2, 3] Yu Yan,[2, 3] Xuezhong Ruan,[2, 3] Yao Li,[2, 3] Hui Zhang,[4, 5] Lin Li,[4, 5] Liang He,[2, 3] Jing Wu,[10, 11, *] Rong Zhang[3], Hongming Weng,[6, 7, 8, *] Changgan Zeng,[4, 5, *] and Yongbing Xu[1, 2, 3, 11, *]

[1]School of Integrated Circuits, Nanjing University, Suzhou 215163, China

[2]State Key Laboratory of Spintronics Devices and Technologies, Nanjing University, Suzhou 215163, China

[3]Jiangsu Provincial Key Laboratory of Advanced Photonic and Electronic Materials, School of Electronic Science and Engineering, Nanjing University, Nanjing 210093, China

[4]International Center for Quantum Design of Functional Materials (ICQD), Hefei National Laboratory for Physical Sciences at the Microscale, and Synergetic Innovation Center of Quantum Information and Quantum Physics, University of Science and Technology of China, Hefei, Anhui 230026, China

[5]CAS Key Laboratory of Strongly-Coupled Quantum Matter Physics, and Department of Physics, University of Science and Technology of China, Hefei, Anhui 230026, China

[6]Beijing National Research Center for Condensed Matter Physics, Institute of Physics, Chinese Academy of Sciences, Beijing 100190, China

[7]School of Physical Science, University of Chinese Academy of Sciences, Beijing 100049, China

[8]Songshan Lake Materials Laboratory, Dongguan, Guangdong 523808, China

[9]Department of Chemistry, University of Pennsylvania, Philadelphia, PA, 19104-6323, USA

[10]School of Integrated Circuits, Guangdong University of Technology, Guangzhou 510006, China

[11]York-Nanjing International Joint Center in Spintronics, School of Physics, Engineering and Technology, University of York, York YO10 5DD, UK

*Authors to whom correspondence should be addressed: jing.wu@gdut.edu.cn, hmweng@iphy.ac.cn, cgzeng@ustc.edu.cn and ybxu@nju.edu.cn

[#]These authors contributed equally: Xianyang Lu, Zhiyong Lin and Hanqi Pi contributed equally to this work

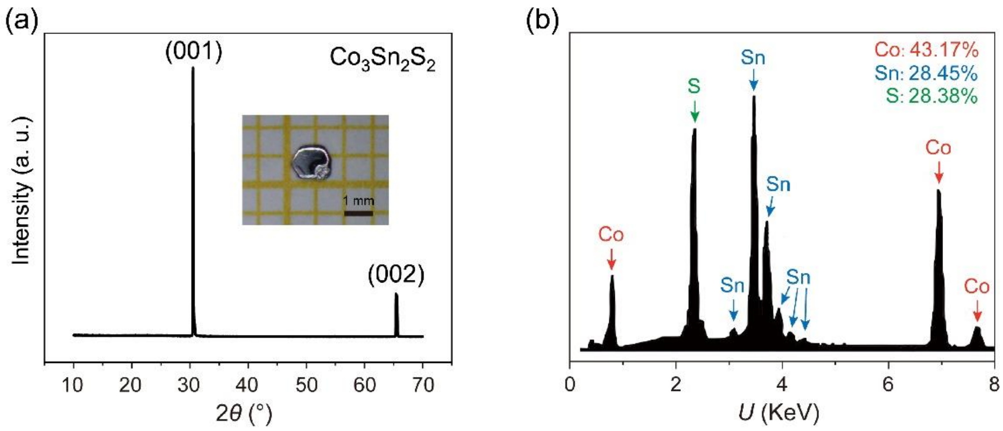

Supplementary Figure 1 | XRD and EDS measurements of $Co_3Sn_2S_2$ crystal. **a**, The measured XRD result of the $Co_3Sn_2S_2$ crystal showing sharp (001) and (002) peaks. Inset is the photo image of the sample **b**, The measured EDS result of the $Co_3Sn_2S_2$ crystal showing the composition of Co:Sn:S is about 3:2:2.

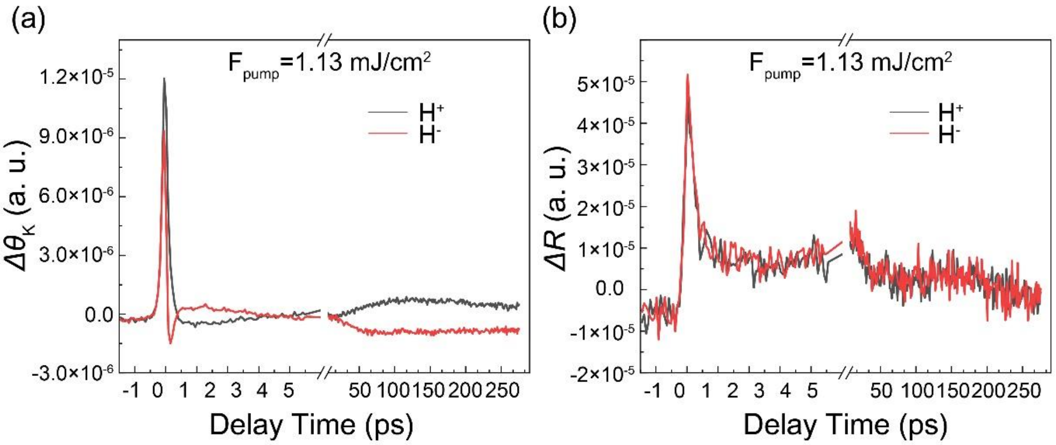

Supplementary Figure 2 | Measured transient Kerr signal and reflectivity change under opposite applied magnetic fields. a, The measured Kerr signal under opposite applied magnetic fields $\Delta\theta_K(\tau, M)$ (black curve) and $\Delta\theta_K(\tau, -M)$ (red curve). The transient Kerr rotation change $\Delta\theta_K(\tau)$ is defined in the following as the asymmetric part, changing with the field direction $\Delta\theta_K(\tau) = (\Delta\theta_K(\tau, M) - \Delta\theta_K(\tau, -M))/2$. **b**, The measured reflectivity change under opposite applied magnetic fields show no difference. The pump fluence is 1.13 mJ/cm^2.

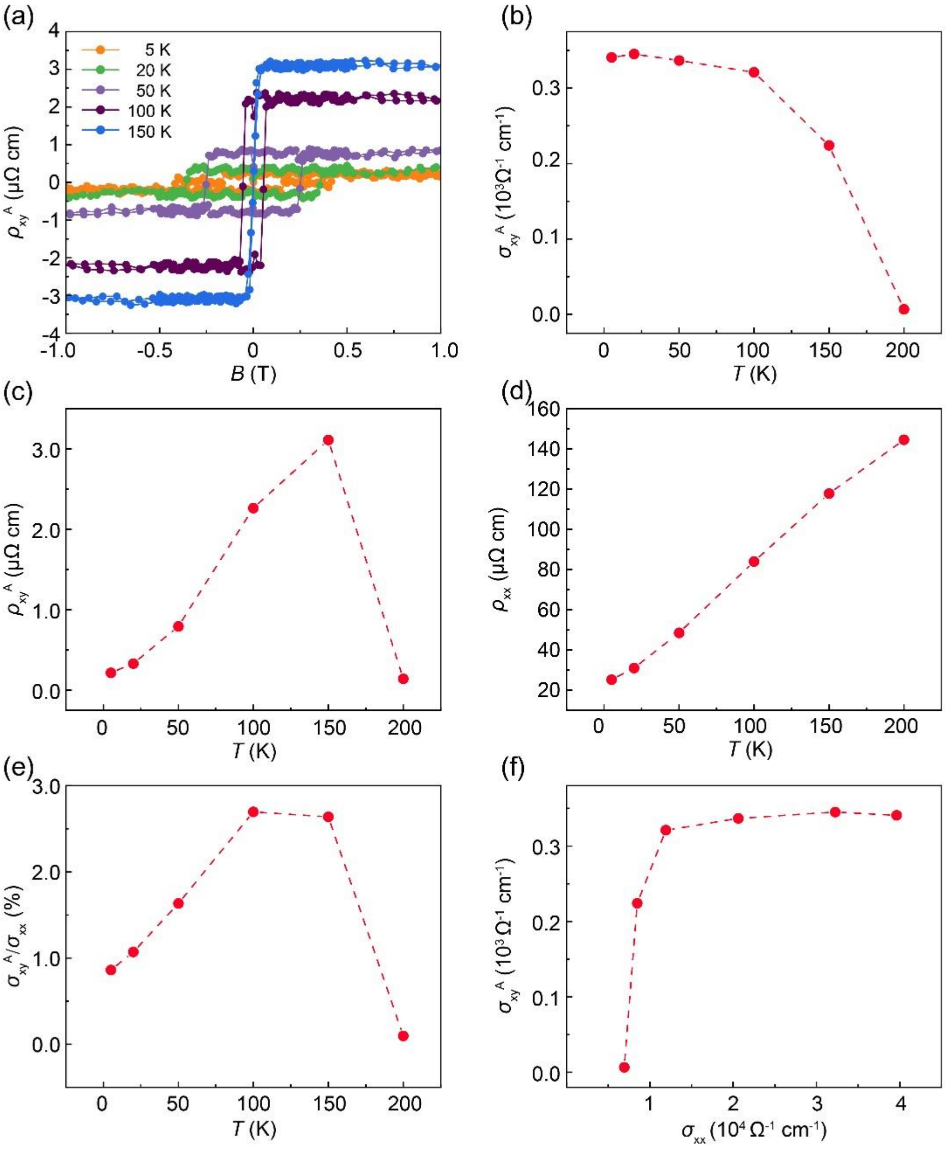

Supplementary Figure 3 | Hall data of $Co_3Sn_2S_2$ crystal. **a**, Hall resistivity ρ_{xy}^{A} measured at different temperatures. **b**, Temperature dependence of the anomalous Hall conductivity σ_{xy}^{A} at zero magnetic field. **c**, Temperature dependence of the anomalous Hall resistivity ρ_{xy}^{A}. The peak around 150 K is consistent with the observation in ref. [3]. **d**, Temperature dependence of the longitudinal resistivity ρ_{xx} at zero magnetic field. **e**, Temperature dependence of the anomalous Hall angle $\sigma_{xy}^{A}/\sigma_{xx}$ at zero magnetic field. **f**, The anomalous Hall conductivity σ_{xy}^{A} as a function of the longitudinal charge conductivity σ_{xx}.

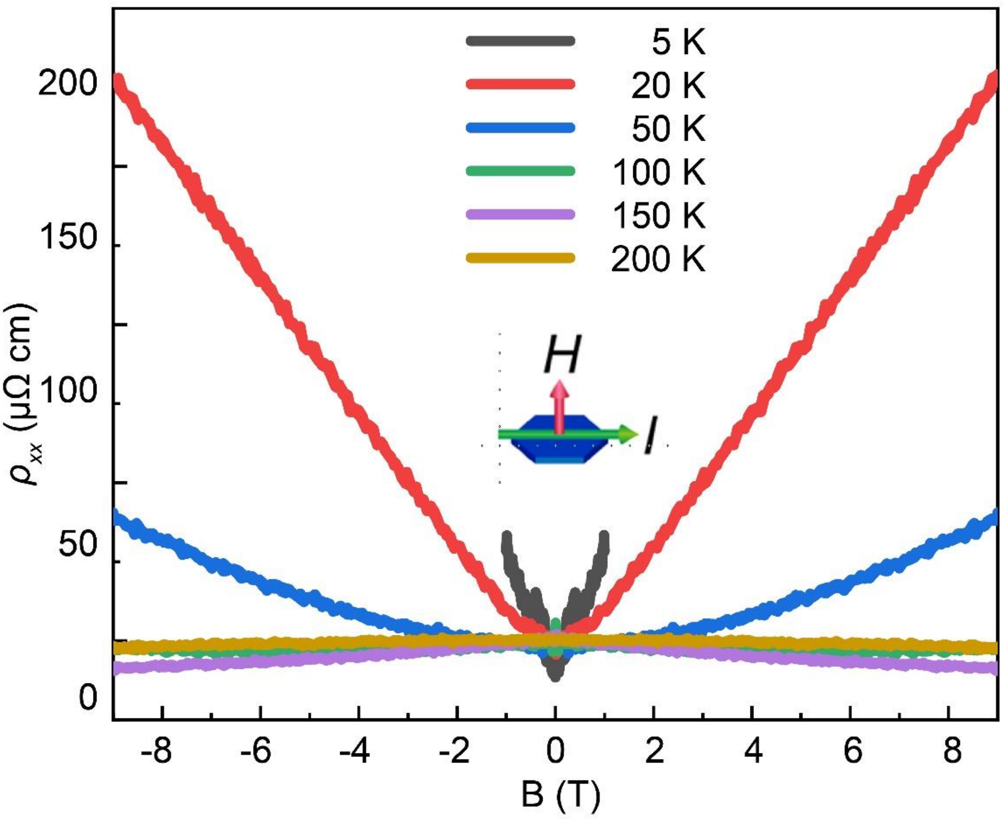

Supplementary Figure 4 | Magnetoresistance of $Co_3Sn_2S_2$ crystal. Magnetoresistance were measured at 5, 20, 50, 100, 150 and 200 K for the applied magnetic field perpendicular to the *ab* kagome plane.

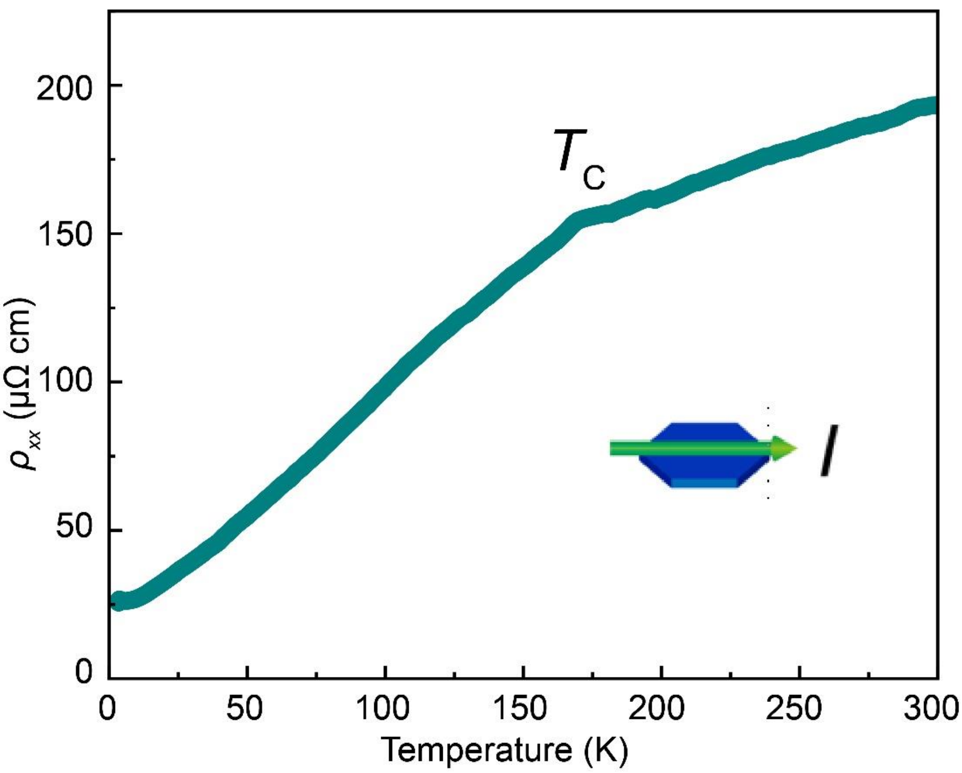

Supplementary Figure 5 | Longitudinal resistance measurement. Temperature dependence of the longitudinal resistivity ρ_{xx} was measured at zero magnetic field. The Curie temperature (~175 K) can be deduced from the kink of the ρ-T curve.

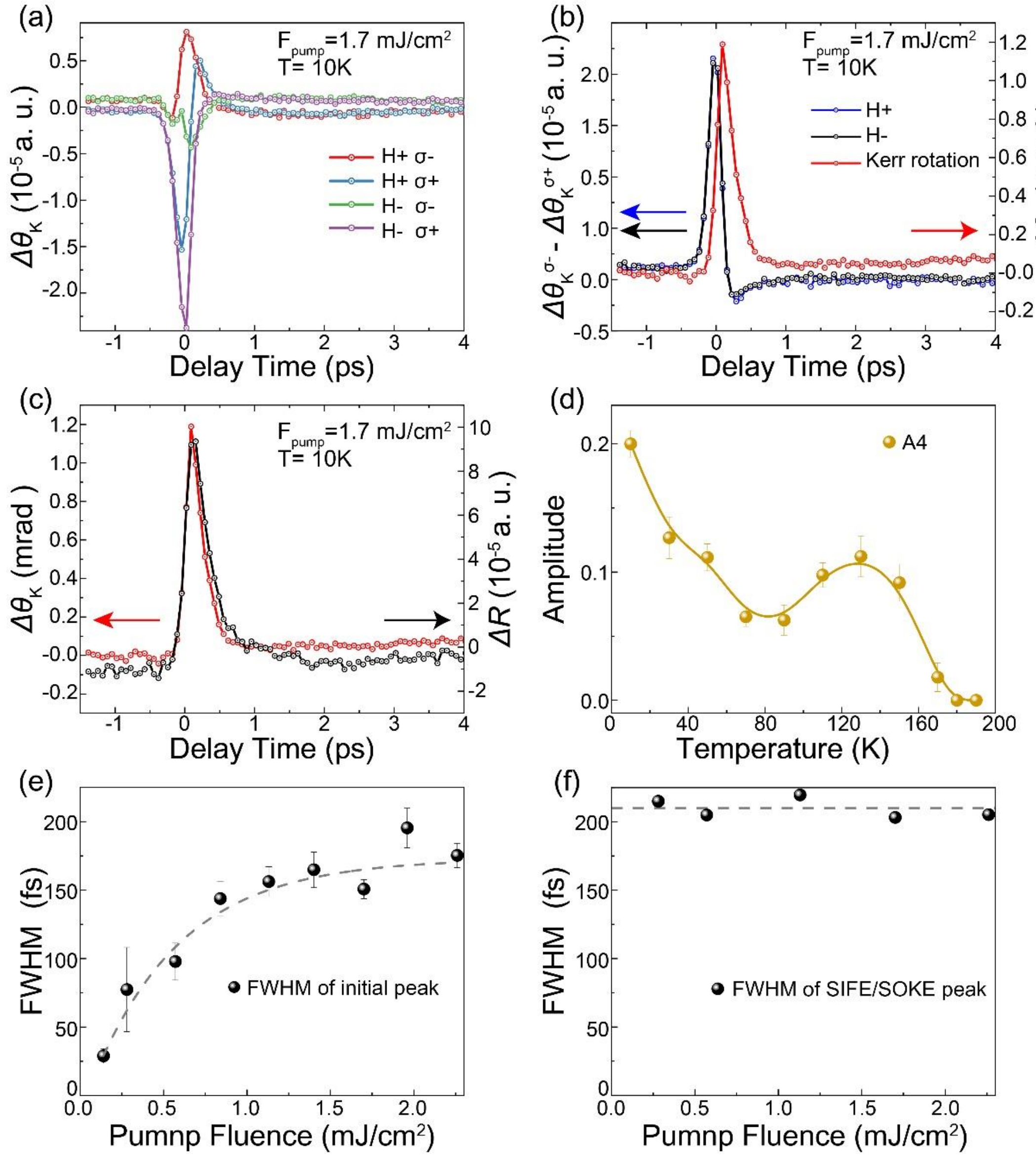

Supplementary Figure 6 | Analysis of the initial peak in measured time-resolved magneto-optic Kerr rotation. a, The measured transient MOKE signal change with different combinations of the applied magnetic field direction (positive H^+ and negative H^-) and the pump helicity (left handed σ^+ and right handed σ^-). **b**, The differential TR-MOKE curves with respect to the pump helicity under the same direction of the applied magnetic field (blue and black) and the transient Kerr rotation curve (red). **c**, The transient Kerr rotation (red) and the transient reflectivity change (black). **d**, The amplitude of the initial peak from time-resolved Kerr rotation A4 as a function of temperature **e**, FWHM of the initial peak from time-resolved Kerr rotation. **f**, FHWM of SIFE/SOKE peak.

Supplementary Note 1. Origin of the initial peak in time-resolved magneto-optic Kerr rotation

To investigate the origin of the initial peak in the TR-MOKE results around zero delay, we firstly consider the contribution of the specular inverse Faraday effect (SIFE) and specular optical Kerr effect (SOKE). These coherent third-order effects only occur during the period of pump excitation. The transient MOKE change with different combinations of the applied magnetic field direction (positive $H^{+}$and negative H^{-}) and the pump helicity (left handed σ^{+} and right handed σ^{-}) is shown in Supplementary Fig. 6a. The pump helicity-induced effect dominates around zero delay can be clearly distinguished, disappearing within 0.5 ps. The differential TR-MOKE curves with respect to the pump helicity under the same direction of the applied magnetic field are plotted in Supplementary Fig. 6b. The SIFE/SOKE peak is illustrated centered at zero delay and this peak coincides for opposite applied magnetic field. A distinct delay (~130 fs) between the initial peak from the time-resolved Kerr rotation and the SIFE/SOKE is revealed. The full width of half maximum (FWHM) of the initial peak from TR-MOKE and the FWHM of the SIFE/SOKE peak as a function of the pump fluence at 10 K are displayed in Supplementary Fig. 6e and f, respectively. The FWHM of the initial peak from TR-MOKE increases gradually with the pump fluence and approaches a saturated value, while the FHWM of the SIFE/SOKE peak remains almost unchanged at ~210 fs. Therefore, we can safely exclude the contribution of SIFE/SOKE to the initial peak in TR-MOKE. Additionally, in a simplified picture, SIFE/SOKE originates from the transfer of angular momentum from circularly polarized photons to electronic orbits[1,2]. However, in our measurements, the initial peak

in TR-MOKE does not depend on the pump polarization, indicating that the SIFE/SOKE contribution can be dismissed a priori.

Interestingly, as shown in Supplementary Fig. 6c, the rising edge of the initial peaks from TR-MOKE and TR-R coincide perfectly. This indicates that the initial peak is determined by the state occupation upon pump excitation. In Supplementary Fig. 6d, the amplitude of the initial peak from TR-MOKE as a function of temperature is plotted (from the fitting results in Fig. 2 in the main text). Overall, the amplitude decreases with increasing temperature and completely vanishes when the temperature reaches the Curie temperature. It is noted that the observed peak around 130 K corresponds to the peak observed in the ZFC curve in Fig. 1h and in Ref. 3. Although the underlying mechanism of this anomalous magnetic transition is still unclear, the amplitude of the initial peak from time-resolved Kerr rotation is highly dependent on the macroscopic magnetization. Thus, the initial peak from time-resolved Kerr rotation can be attributed to the so-called dichroic bleaching or state blocking effect[4,5]. This effect can be interpreted as a breakdown of the proportionality between the magnetization and the Voigt vector that is the basis of magneto-optics due to the consequence of the out-of-equilibrium character of the electron system immediately after the femtosecond excitation. To verify the bleaching and state-blocking effect, a spectral dependence in the magneto-optical response would be expected[6]. Unfortunately, although the spectral dependence of the magneto-optical effect in $Co_3Sn_2S_2$ has been studied recently, the photon energy used was very low (terahertz to 1 eV)[7]. The spectral dependence of the magneto-optical response around the wavelength of the probe pulse (400 nm) still

deserves further investigation. Nevertheless, as this bleaching or state-blocking effect only lasts during the first hundreds of femtoseconds upon pump excitation, the subsequent magneto-optical response predominantly reflect the genuine spin dynamics in $Co_3Sn_2S_2$.

Supplementary Note 2. Details of data fitting process

We carefully fitted our experimental results, and extensive effort has been made to avoid any artifact or over-interpretation during the fitting process. As discussed in the main text, numerically fitting three competing magnetization dynamic processes (and the initial peak) is difficult because each component has a characteristic enhancement (demagnetization) time and a corresponding relaxation time. If we fit all these three pairs of time constants as free variables, the fitting results could be divergent and arbitrary. Therefore, we fixed several parameters correspondingly when processing the pump fluence-dependent results at 10 K.

For the TR-MOKE curves measured at 10 K with various pump fluences, we found that all the curves can be fitted very well with a fixed combination of $\tau_{enh} = 2$ ps, $\tau_{fast} = 300$ fs and $\tau_{slow} = 6$ ps. In Ref. 8,9, by applying the time-resolved angular-resolved photoemission spectroscopy (TR-ARPES), which is surface sensitive, a critical behavior of the demagnetization time is observed with respect to the pump fluence in Ni film. Two unchanged values of the demagnetization time are observed below or above the critical pump fluence. Therefore, the demagnetization dynamics observed by TR-MOKE with a continuously varied demagnetization time as a function

of the pump fluence can be interpreted as an integration of the magnetic response over the penetration depth of the probe light. From the sample surface the pump fluence is attenuated gradually within the penetration depth. Here, in this study, the three magnetization dynamic components can be qualitatively distinguished in Fig. 1k. Although whether (part of) these components observed in $Co_3Sn_2S_2$ have a critical behavior with respect to the pump fluence as similar as Ni is unknown, we believe the current fitting method to recognize each magnetization dynamic components is scientifically sound. Indeed, the *precise* values of the demagnetization (enhancement) time constant and their pump fluence dependence are desirable (and call for the further investigation). However, the three distinct characteristic time constants $\tau_{enh} = 2$ ps, $\tau_{fast} = 300$ fs and $\tau_{slow} = 6$ ps can sufficiently provide information for the following discussion.

For the TR-MOKE curves measured as a function of temperature, as shown in Fig. 2a, the assumption of unchanged characteristic times for each magnetization dynamic component is not reasonable because temperature variation would affect both the macroscopic magnetization and the topological properties. In this case, we choose the temperature-dependent TR-MOKE results with a low pump fluence of 0.57 mJ/cm^2 for investigate. As shown in Supplementary Fig. 7, the TR-MOKE curves measured at 10 K show that the A3 component (slow demagnetization) is considerably small compared to the other two components under such low pump fluence. Thus, we ignore the A3 component when fitting the temperature dependent TR-MOKE curves when the temperature is lower than 90 K. All the other parameters, including τ_{enh}, τ_{fast} and

τ_{slow}, are open for fitting. We note that the A3 amplitude below 90 K as shown in Fig. 2c should be a very small value rather than absolute zero. However, this simplification does not affect the key observation of the transition around 110 K. The fitted values of τ_{enh} and τ_{fast}, as shown in Supplementary Table. 3, are close to the values used in the pump fluence-dependent results at 10 K, which demonstrates again the validity of the fitting method mentioned above.

The fitting values of all the parameters for the pump fluence-dependent at 10 K and temperature-dependent at 0.57 mJ/cm^2 measurements are included in Supplementary Table 2 and 3, respectively.

Supplementary Note 3. Potential mechanisms for the ultrafast magnetization enhancement

Ultrafast laser-stimulated magnetization enhancement on a 100 ps timescale was observed in the diluted magnetic semiconductor GaMnAs[10]. This photoenhanced ferromagnetism was attributed to the collective ordering to the *p-d* exchange interaction between photoexcited holes and Mn spins. Two key pieces of evidence for this Mn-hole correlation are the experimentally observations of the peak near 20 K of the photoenhanced ferromagnetism and the observed enhanced magnetization even above the Curie temperature. However, in $Co_3Sn_2S_2$, the magnetization enhancement is monotonously decreasing with increasing temperature. Also, no transient Kerr rotation is observed in $Co_3Sn_2S_2$ when the temperature is higher than the Curie temperature, regardless of the pump fluence. Therefore, this nonthermal 3d transition metal-hole

exchange correlation-induced magnetization is not the mechanism responsible for our results.

It has been reported that ultrafast photoinduced insulator-metal transitions can induce ultrafast antiferromagnet-ferromagnet transitions in perovskite-type $Gd_{0.55}Sr_{0.45}MnO_3$[11]. Firstly, the photocarriers melt the charge-ordered and orbit-ordered insulator phase, and the metallic state is formed within the time resolution of ~200 fs. Meanwhile, the charge excitation destructs the charge ordering and causes the charges to become delocalized. Secondly, in the delocalized state, the manipulated double-exchange interaction causes the spins to align ferromagnetically. In $Co_3Sn_2S_2$, although theoretical investigations indicate that the third-neighbor exchange coupling via Co-Sn-Co dominates when temperature is below the Weyl nodes annihilation temperature[12], this *switched-on* exchange-based ultrafast magnetization enhancement is extremely fast with a characteristic time of 20 fs for $Gd_{0.55}Sr_{0.45}MnO_3$ (corresponding to the double exchange energy of ~0.2 eV). The calculated effective energy of the third-neighbor exchange in $Co_3Sn_2S_2$ is ~0.03 eV[12], which corresponds to a characteristic time of ~133 fs. However, the characteristic time of our observed magnetization enhancement is ~2 ps, which is larger than 133 fs by an order of magnitude. Also, this exchange-coupling-induced magnetization enhancement cannot lasts for a long time (decay in ~10 ps for $Gd_{0.55}Sr_{0.45}MnO_3$), while the magnetization enhancement in $Co_3Sn_2S_2$ shows a long relaxation time of over 100 ps. From a microscopic view, unlike the insulating $Gd_{0.55}Sr_{0.45}MnO_3$, $Co_3Sn_2S_2$ is a semimetal that does not exhibit (weak) charge ordering. Thus, the laser pulse cannot stimulate the destruction of charge ordering and

subsequently tuning of the exchange coupling. Therefore, the observation of magnetization enhancement in $Co_3Sn_2S_2$ cannot be attributed to photoinduced (charge delocalized) exchange coupling modulation, although the exact evolution of exchange coupling upon the laser stimulation is worth further investigation.

Ultrafast magnetization enhancement was also observed in metallic multilayers driven by superdiffusive spin current[13]. However, this effect can be safely excluded in our experiments as there is no adjacent magnetic compound.

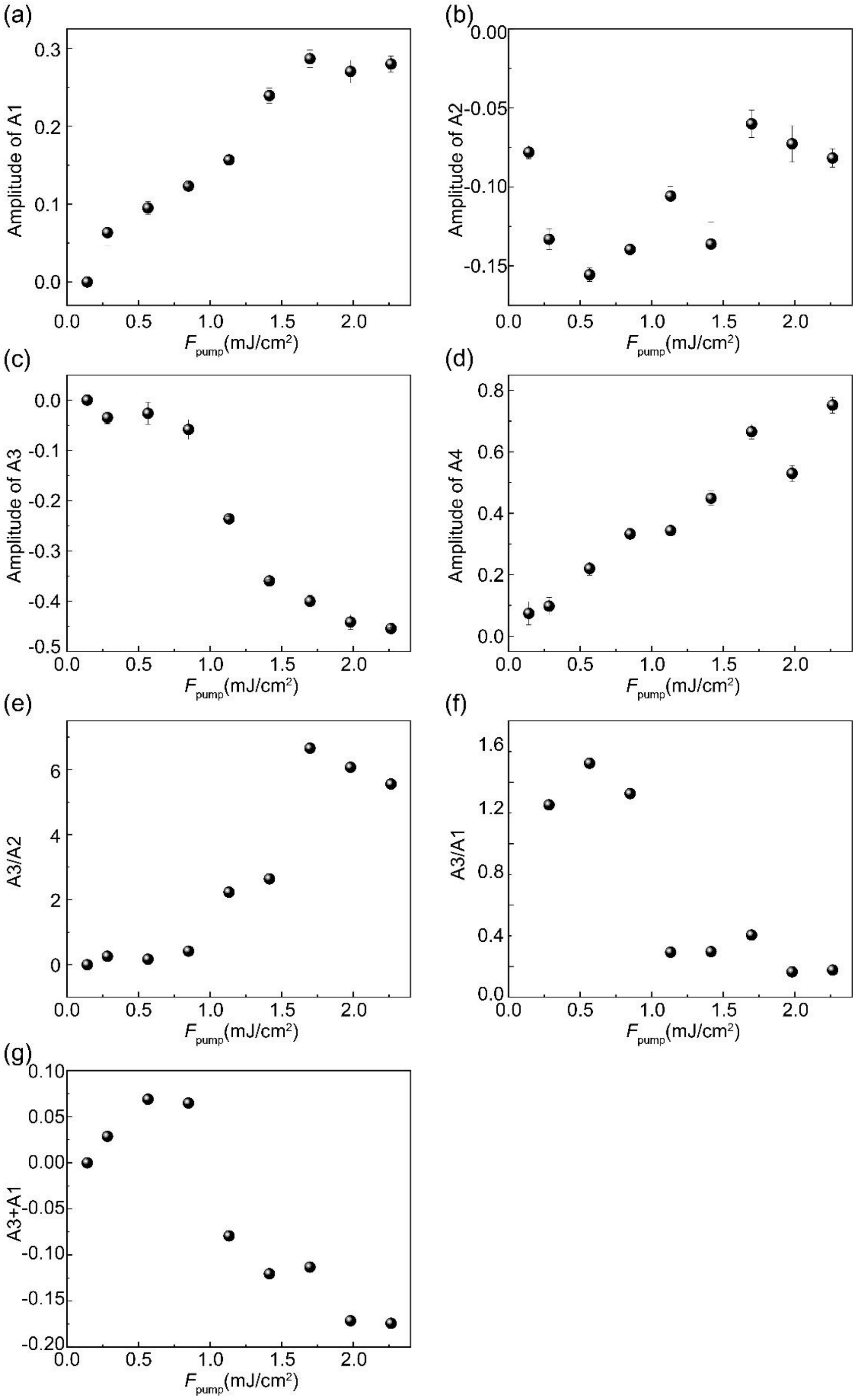

Supplementary Figure 7| Fitted amplitudes of the magnetization dynamic components. a-d, Amplitudes of the components A1, A2, A3 and A4 as a function of pump fluence at 10 K, respectively. **e-f**, The ratios of A3/A2 and A3/A1 as a function of pump fluence at 10 K, respectively. **g**, A1+A3 as a function of pump fluence at 10 K.

Supplementary Note 4. Electronic transition excited by the pump light

The optical spectra can provide significant insights into electronic transitions under pump beam excitation. Unfortunately, direct optical spectra measurements on $Co_3Sn_2S_2$ are limited. To our knowledge, the most relevant published work is Ref [14]. Although the optical spectra in that study were meticulously measured and supported by first-principle calculations, the highest applied photon energy is 1 eV, much lower than the photon energy of 1.55 eV used here. Nonetheless, we can refer to this work for some information on electronic transitions.

As depicted in Fig. 3c and Supplementary Fig. 7 in Ref [14], while the excitation light energy is increasing, the potential for the corresponding interband transitions also grows. Therefore, by applying pump light with a photon energy of 1.55 eV, more bands would be excited across a broader k-space. The excited electrons are far above the Fermi level and much higher than the Weyl nodes in the momentum space. This picture is in consistent with that depicted in Ref [15].

Supplementary Note 5. Long demagnetization time in magnetic half-metals

In comparison to ferromagnetic metals, studies on magnetic half-metals[16-18] have demonstrated a distinct long demagnetization time scale which could be of up to 1000 picoseconds (ps).[19] The prominent characteristic of half-metal is that there is a spin-polarized band gap at the Fermi level so that a high spin polarization is presented. Compared to transition metals, it is proposed that this will considerably slow the demagnetization process in half-metals arising from the blocking Elliot-Yafet scattering

process due to intrinsic half-metallic nature. The Elliot-Yafet spin-flip provides an interaction channel between the electron and spin systems via the band mixing for majority and minority spins. Instead of this direct angular momentum transfer, in half-metals the spin-lattice interaction plays an overwhelming role which is mediated by spin-orbit coupling[17,20] and the time constant of this spin thermalization process is much longer than the normal demagnetization time in transition metals.

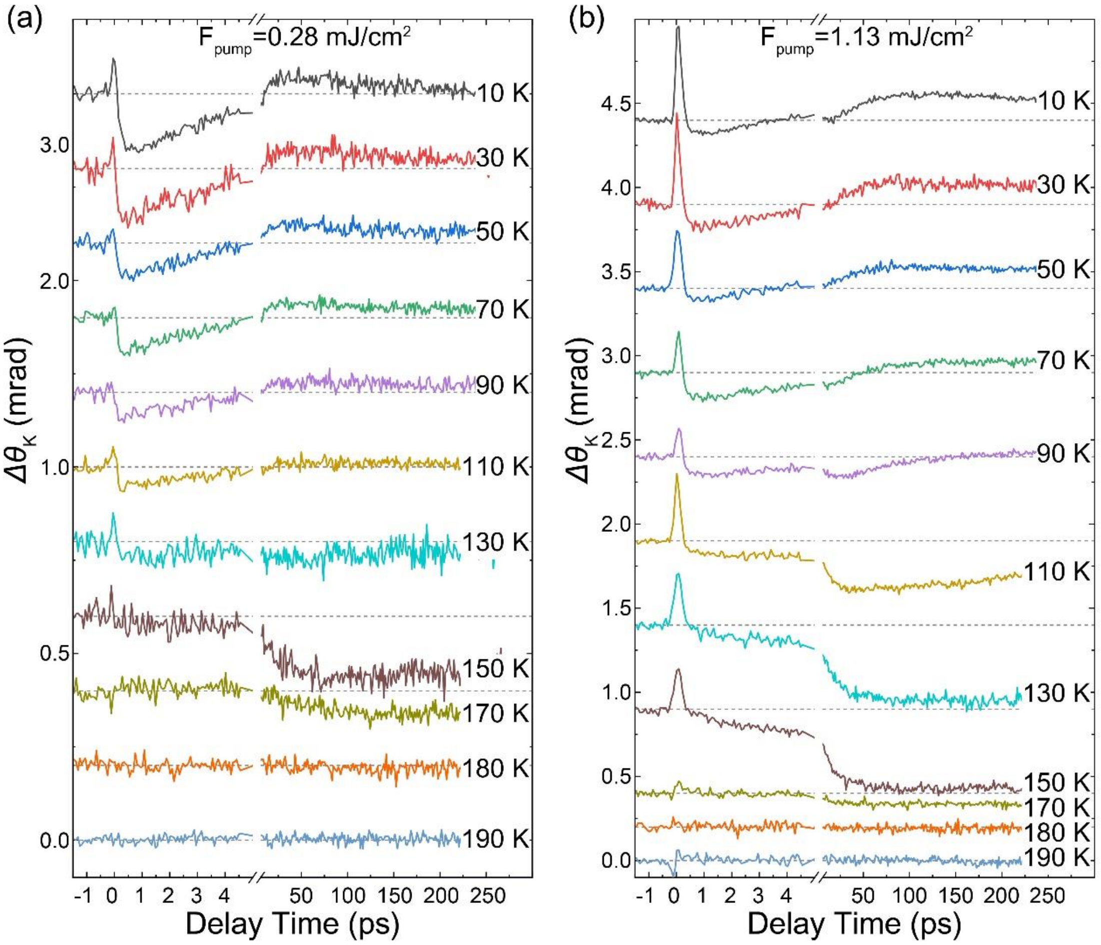

Supplementary Figure 8| Magnetization dynamics of $Co_3Sn_2S_2$ measured as a function of the temperature. a-b, The measured transient Kerr rotation of $Co_3Sn_2S_2$ at temperature varying from 10 K to 190 K with the pump fluence of 0.28 mJ/cm^2 and 1.13 mJ/cm^2, respectively. Circles are experimental data and the solid lines are the fitted curves.

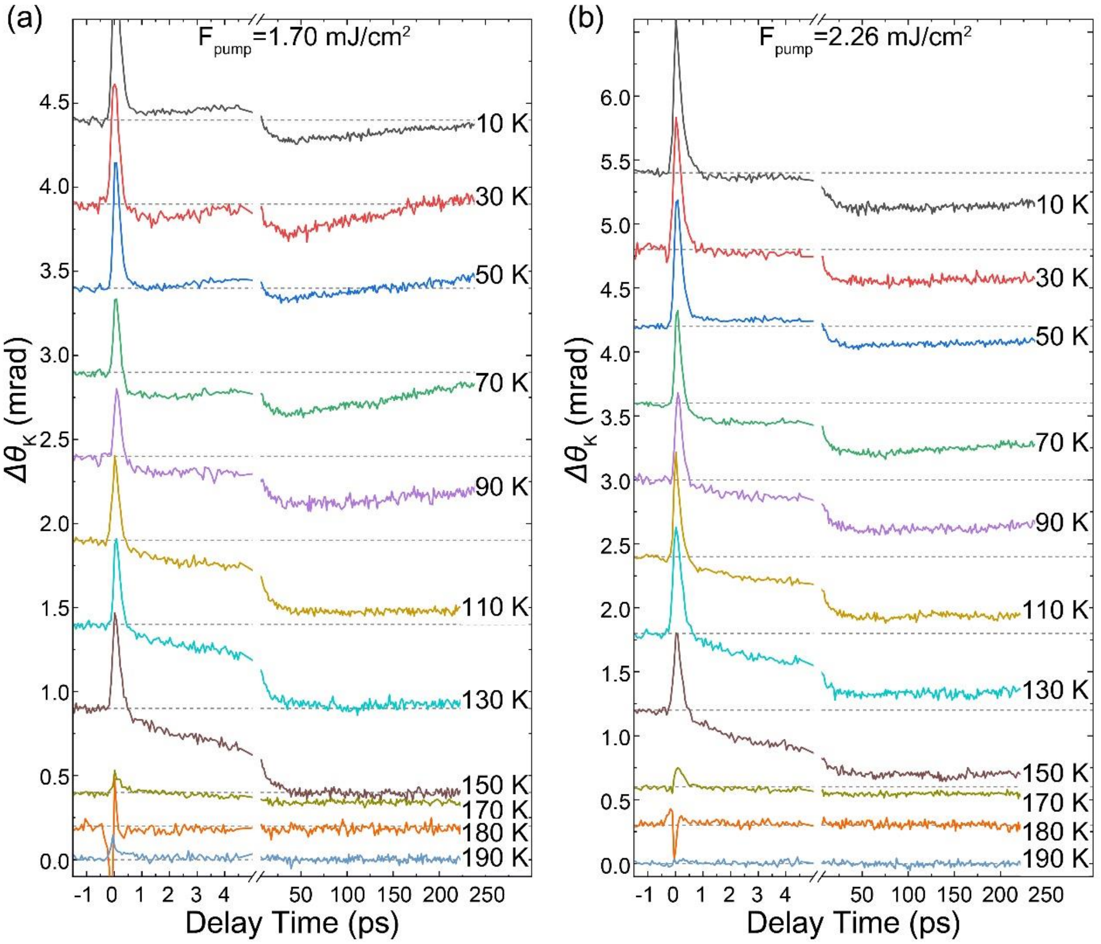

Supplementary Figure 9| Magnetization dynamics of $Co_3Sn_2S_2$ measured as a function of the temperature. a-b, The measured transient Kerr rotation of $Co_3Sn_2S_2$ at temperature varying from 10 K to 190 K with the pump fluence of 1.70 mJ/cm^2 and 2.26 mJ/cm^2, respectively. Circles are experimental data and the solid lines are the fitted curves.

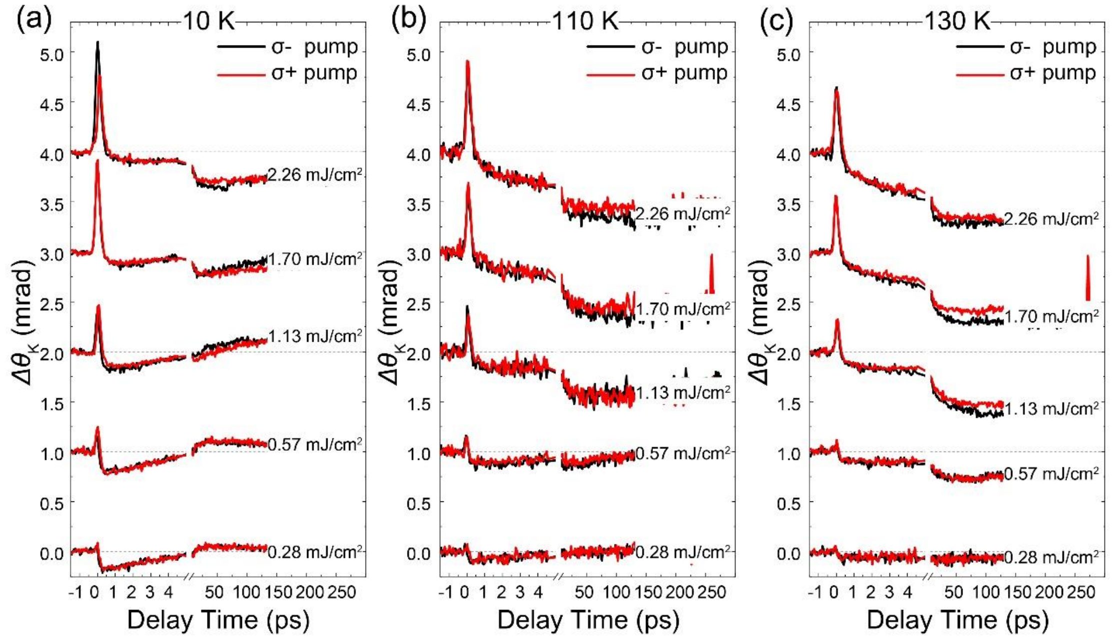

Supplementary Figure 10| Magnetization dynamics of $Co_3Sn_2S_2$ measured as a function of the pump polarization. a-c, The measured transient Kerr rotation of $Co_3Sn_2S_2$ stimulated by the left-handed and right handed pump pulses at temperature of 10 K, 110 K and 130 K respectively. The pump fluence is varying from 0.28 mJ/cm^2 to 2.26 mJ/cm^2

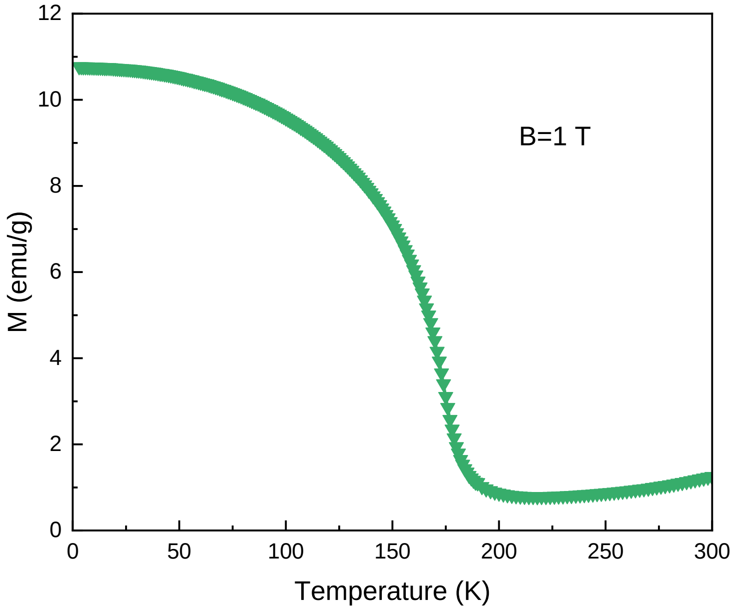

Supplementary Figure 11| Field cool measurements on temperature dependence of magnetization, the applied filed is 1 Tesla along c axis.

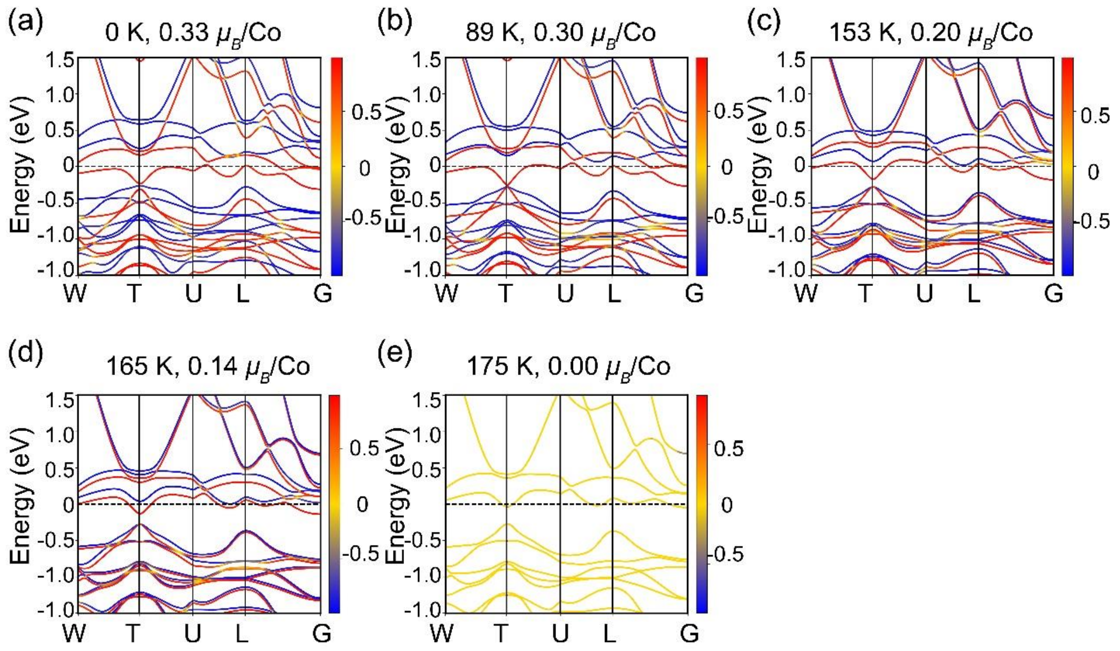

Supplementary Figure 12| The band structure along high-symmetry paths. a-d, the temperature (magnetization) dependent band structure along high-symmetry paths**.** The bands with +1/2, 0 and -1/2 spin polarization are coloured in red, yellow and blue, respectively. **e**, Band structure in the paramagnetic state.

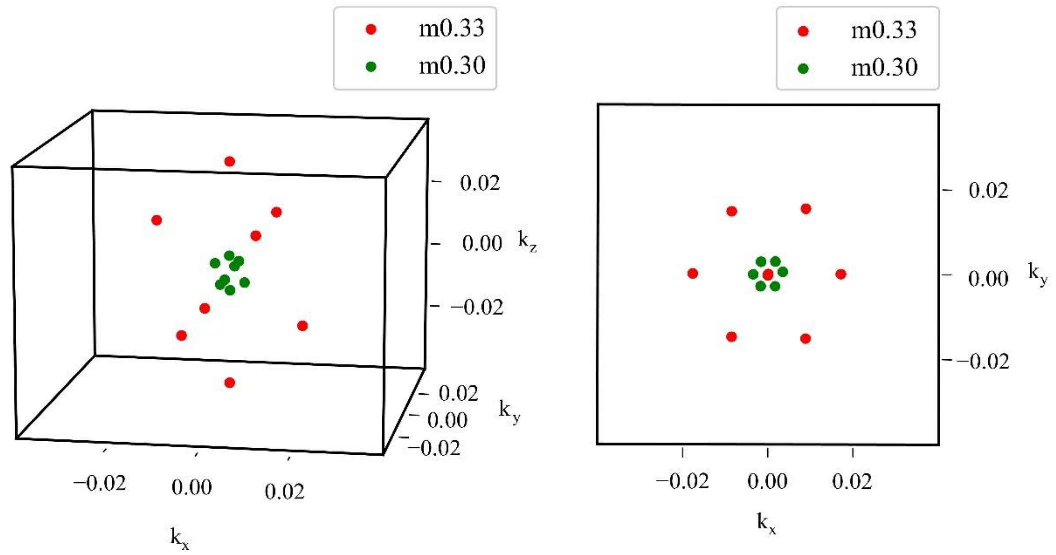

Supplementary Figure 13| Four pairs of type-II Weyl nodes in k space. Cartesian coordinates of the four pairs of type-II Weyl nodes in k space. Zero point corresponds to the Γ point. Red and green dots represent the location when the local magnetic moment on Co is 0.33 μ_B and 0.30 μ_B, respectively.

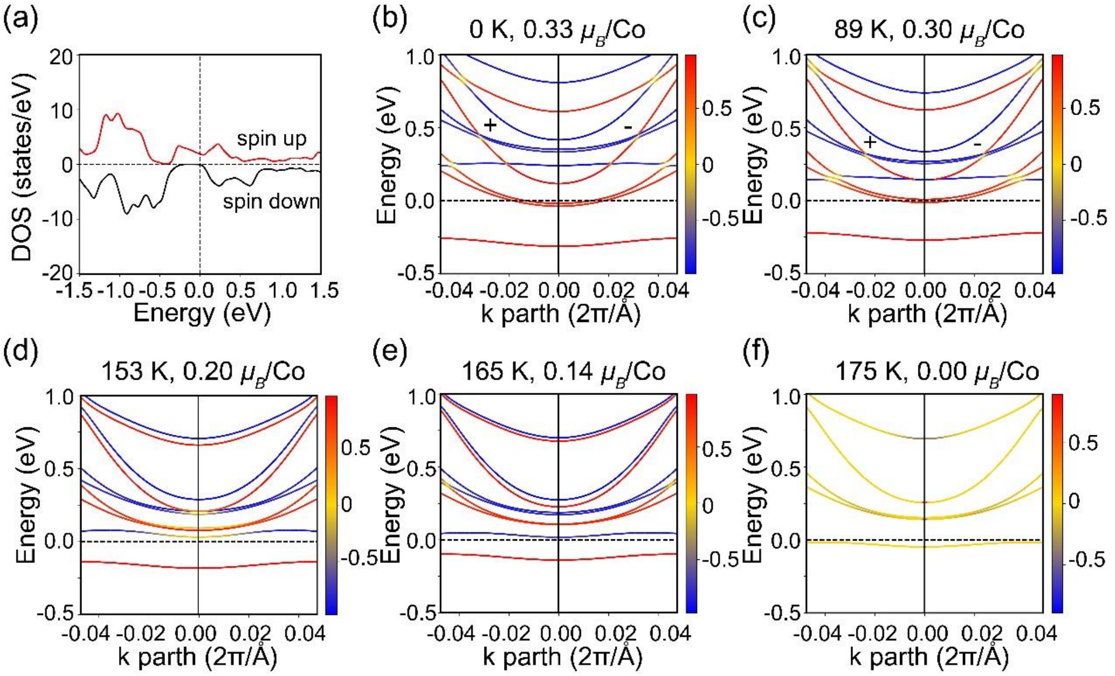

Supplementary Figure 14| Temperature-dependent band structures around Weyl nodes by first-principle calculation. a. Spin dependent density of states at ground state. **b-f.** Band structures along the momentum path passing through a pair of Weyl nodes with opposite chirality and the Γ point at T = 0, 89, 153, 165, and 175 K. The bands with +1/2, 0, and −1/2 spin polarization are colored in red, yellow, and blue, respectively.

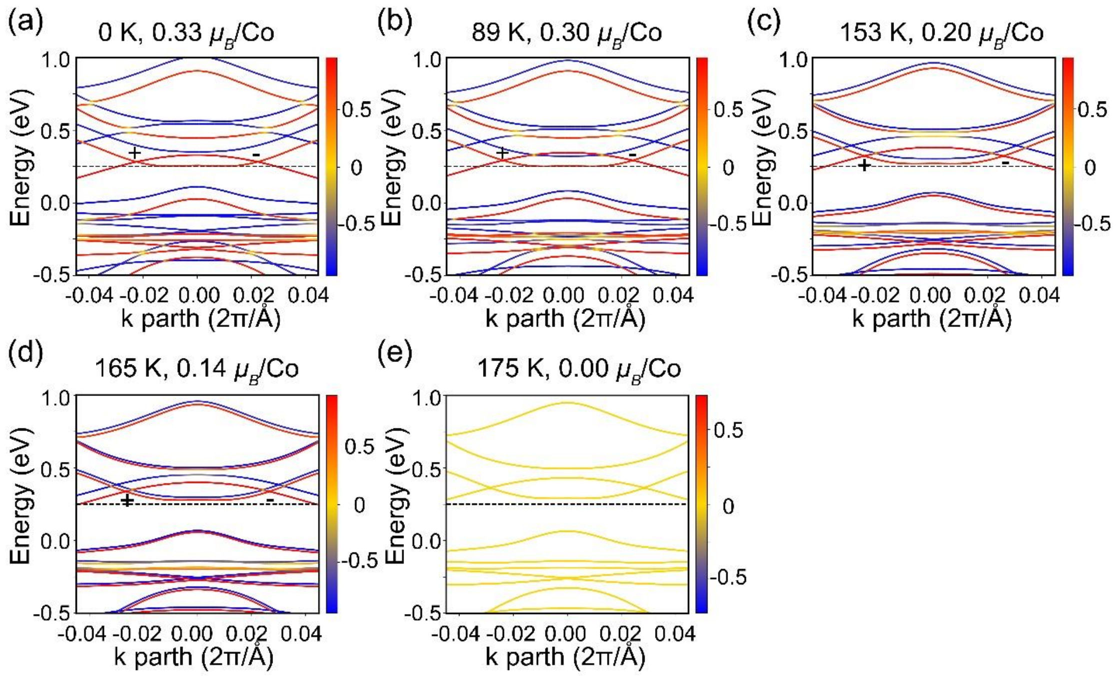

Supplementary Figure 15| Temperature-dependent band structures around Weyl nodes at 60 meV by first-principle calculation. (a)-(e) Band structures along the momentum path passing through a pair of Weyl nodes with opposite chirality and the Γ point at T = 0, 89, 153, 165, and 175 K. The bands with +1/2, 0, and −1/2 spin polarization are colored in red, yellow, and blue, respectively.

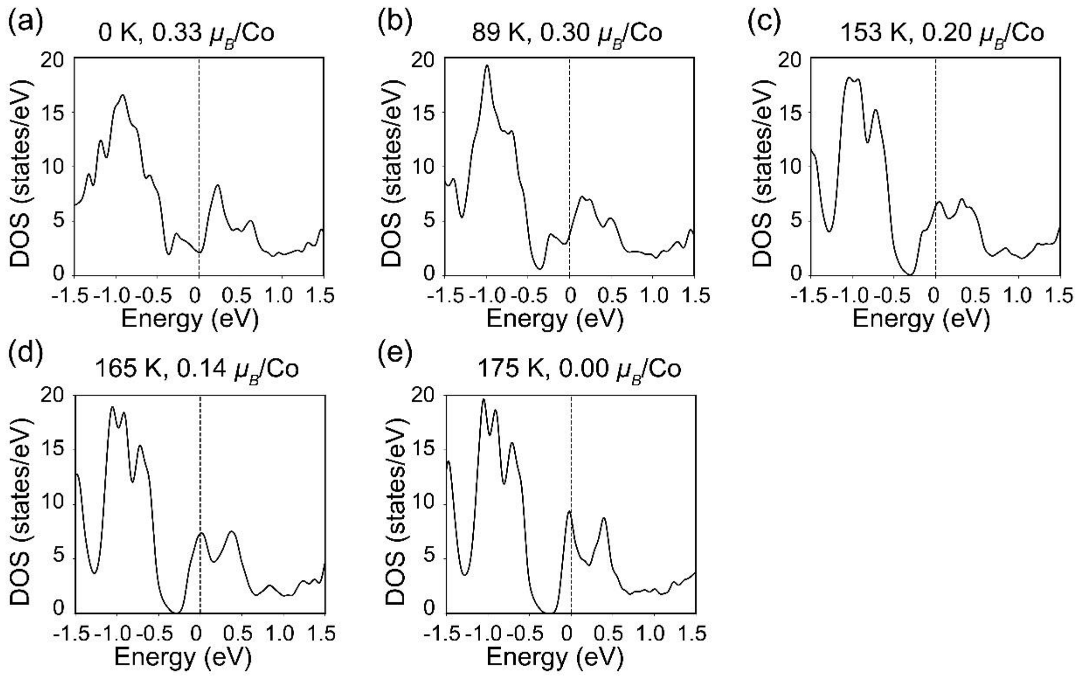

Supplementary Figure 16| Density of states as a function of temperature. (a)-(e) The calculated density of states of $Co_3Sn_2S_2$ at T = 0, 89, 153, 165, and 175 K. The ferromagnetism greatly suppresses the density of state near the Fermi level.

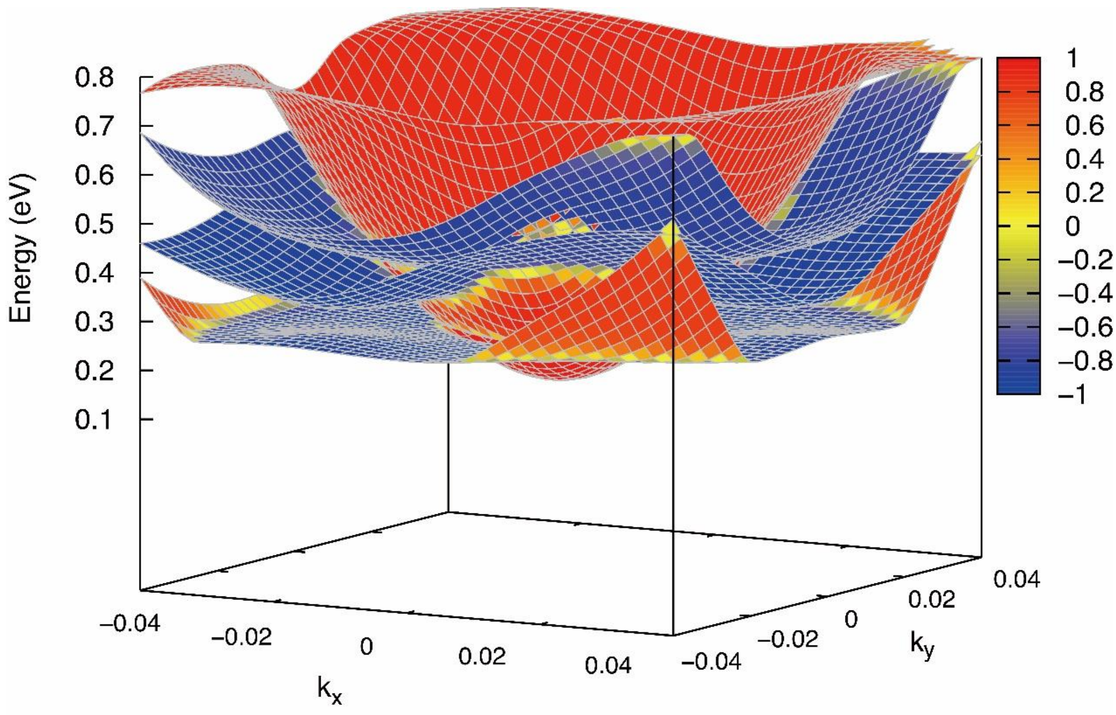

Supplementary Figure 17| Spin-up polarized pocket under the Weyl nodes. Three-dimensional perspective views of the band structures around the Weyl nodes (k_z = 0.015). The bands with +1/2, 0 and -1/2 spin polarization are coloured in red, yellow and blue, respectively.

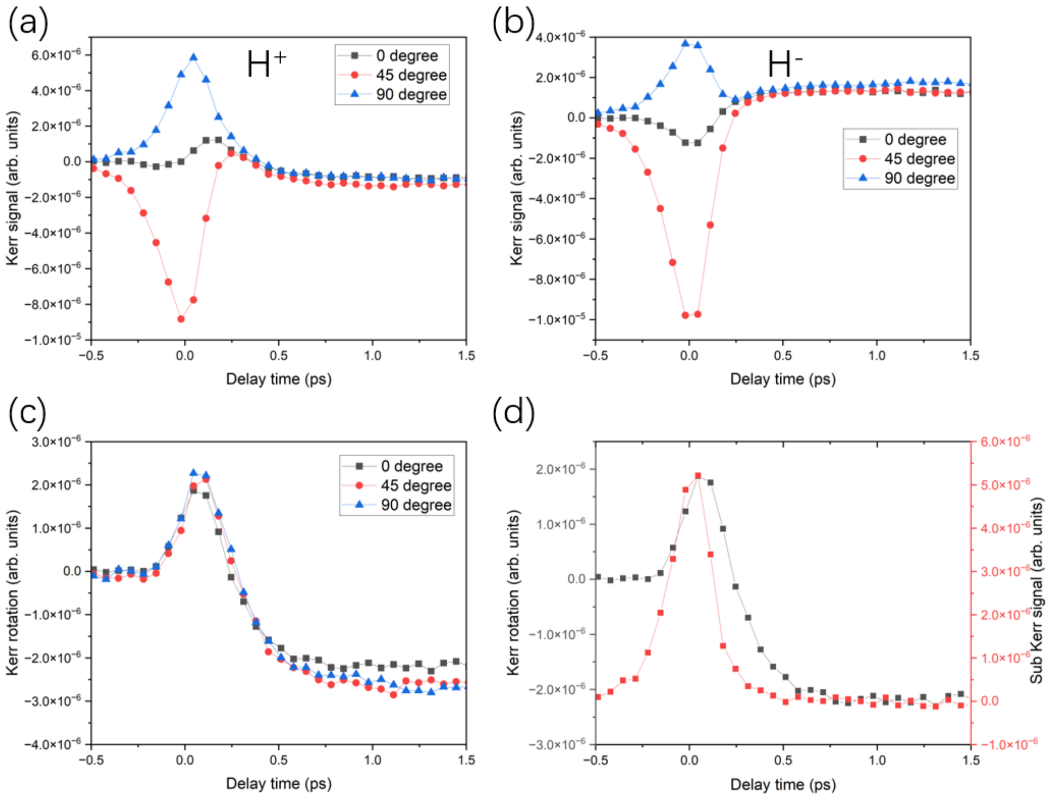

Supplementary Figure 18 a, The measured transient MOKE signal with different pump beam polarization direction under positive applied magnetic field H^{+}. **b**, The measured transient MOKE signal with different pump beam polarization direction under positive applied magnetic field H^{-}. **c**, The transient Kerr rotation curves with different pump beam polarization direction **d**, the differential curve (red) of the Kerr signals for the pump beam is s and p polarized under the same external field $\Delta\theta_K(\tau, M, 0^{\circ}) - \Delta\theta_K(\tau, M, 90^{\circ})$ and the transient Kerr rotation curve (black).

Supplementary Note 6. Pump beam polarization-dependent measurements of the initial peak in time-resolved magneto-optic Kerr rotation

We measured the initial peaks by rotating the linear polarization direction of the pump beam. The probe beam is fixed as p-polarized, and the pump beam was tuned from s polarized (0 degree), 45 degree away from s-polarized (45 degree), to p polarized (90 degree). The Kerr signals measured with positive magnetization and negative magnetization, denoted as $\Delta\boldsymbol{\theta}_{\boldsymbol{K}}(\boldsymbol{\tau}, \boldsymbol{M})$ and $\Delta\boldsymbol{\theta}_{\boldsymbol{K}}(\boldsymbol{\tau}, -\boldsymbol{M})$ respectively, are shown in Supplementary Fig. 18(a) and (b). The initial peaks around zero delay are highly dependent on the pump beam polarization direction. They are maximized when the planes of polarization of pump and probe are 45 degree apart. Therefore, as stated in Supplementary Note 1 of the supplementary information, these peaks are attributed to SIFE/SOKE. The transient Kerr rotation changes are obtained as the asymmetric part of the measured Kerr signal with opposite external fields: $\Delta\boldsymbol{\theta}_{\boldsymbol{K}}(\boldsymbol{\tau}) = (\Delta\boldsymbol{\theta}_{\boldsymbol{K}}(\boldsymbol{\tau}, \boldsymbol{M}) - \Delta\boldsymbol{\theta}_{\boldsymbol{K}}(\boldsymbol{\tau}, -\boldsymbol{M}))/\mathbf{2}$, as shown in Supplementary Fig. 18c. The initial peaks, referred to as the A_1 part in the manuscript, remain unchanged with respect to the pump beam polarization direction. We also compared the initial peak in Kerr rotation with the differential curve of the Kerr signals when the pump beam is s-polarized and p-polarized under the same external field: $\Delta\boldsymbol{\theta}_{\boldsymbol{K}}(\boldsymbol{\tau}, \boldsymbol{M}, \mathbf{0}^{\circ}) - \Delta\boldsymbol{\theta}_{\boldsymbol{K}}(\boldsymbol{\tau}, \boldsymbol{M}, \mathbf{90}^{\circ})$, as shown in the red line Supplementary Fig. 18d. It is evident that the A_1 peak of the Kerr rotation (black line in Supplementary Fig. 18d) is significantly delayed compared to the SIFE/SOKE peak, with a delay time of ~130 fs. This observation is consistent with Supplementary Fig. 6b. Therefore, the pump beam polarization-dependent

measurements support our analysis of the initial peak in the manuscript. We note that ellipticity measurements may provide more information about the initial peak, which certainly deserve further study in future work.

Supplementary Table S1. Cartesian coordinate of four pairs of Weyl nodes (Γ point is at $k_x = 0, k_y = 0, k_z = 0$)

	m = 0.33 μ_B/Co		m = 0.30 μ_B/Co	
	+	-	+	-
Three pairs	(0.0083, 0.0144, 0.0154)	(-0.0083, -0.0144, -0.0154)	(0.0016, 0.0027, 0.0029)	(-0.0016, -0.0027, -0.0029)
	(0.0083, -0.0144, 0.0154)	(-0.0083, 0.0144, -0.0154)	(0.0016, -0.0027, 0.0029)	(-0.0016, 0.0027, -0.0029)
	(0.0166, 0.0000, 0.0154)	(-0.0166, 0.0000, -0.0154)	(0.0032, 0.0000, 0.0029)	(-0.0032, 0.0000, -0.0029)
One pair	(0.0000, 0.0000, -0.0334)	(0.0000, 0.0000, 0.0334)	(0.0000, 0.0000, -0.0054)	(0.0000, 0.0000, 0.0054)

Units: 2π/Å

Supplementary Table S2. Fitting parameters for the pump fluence dependent measurements when temperature is 10 K.

Pump fluence (mJ/cm^2)	A1(a. u.)	A2(a. u.)	A3(a. u.)	A4(a. u.)	τ_{enh}(ps)	τ_{r1}(ps)	τ_{fast}(ps)	τ_{r2}(ps)	τ_{slow}(ps)	τ_{r3}(ps)	τ_{r4}(ps)
0.14	0	-0.078 ± 0.004	0	0.074 ± 0.038	-	-	0.3	4.18 ± 0.28	-	-	0.021 ± 0.008
0.28	0.063 ± 0.017	-0.133 ± 0.007	-0.035 ± 0.012	0.098 ± 0.028	2	96.4 ± 8.4	0.3	7.77 ± 0.92	6	43.9 ± 18.1	0.055 ± 0.022
0.57	0.095 ± 0.008	-0.156 ± 0.004	-0.026 ± 0.022	0.219 ± 0.021	2	194.5 ± 13.5	0.3	10.22 ± 2.30	6	35.7 ± 25.1	0.069 ± 0.010
0.85	0.123 ± 0.003	-0.140 ± 0.004	-0.058 ± 0.020	0.332 ± 0.020	2	281.8 ± 8.9	0.3	18.94 ± 3.16	6	40.2 ± 17.4	0.102 ± 0.009
1.13	0.157 ± 0.005	-0.106 ± 0.006	-0.236 ± 0.004	0.343 ± 0.016	2	336.8 ± 16.6	0.3	3.55 ± 0.42	6	42.5 ± 1.6	0.111 ± 0.008
1.41	0.239 ± 0.010	-0.136 ± 0.006	-0.360 ± 0.002	0.449 ± 0.024	2	358.3 ± 8.1	0.3	4.08 ± 2.70	6	115.1 ± 16.6	0.117 ± 0.009
1.70	0.287 ± 0.011	-0.060 ± 0.009	-0.400 ± 0.012	0.666 ± 0.024	2	353.6 ± 10.2	0.3	4.63 ± 1.46	6	251.7 ± 1.6	0.107 ± 0.005
1.98	0.270 ± 0.015	-0.073 ± 0.011	-0.442 ± 0.015	0.529 ± 0.026	2	358.2 ± 11.3	0.3	4.22 ± 2.01	6	460.7 ± 6.5	0.138 ± 0.010
2.26	0.280 ± 0.010	-0.082 ± 0.006	-0.454 ± 0.001	0.752 ± 0.026	2	353.1 ± 9.3	0.3	3.88 ± 1.57	6	557.7 ± 5.1	0.124 ± 0.006

Supplementary Table S3. Fitting parameters for the temperature dependent measurements when the pump fluence is 0.57 mJ/cm^2.

Temperature (K)	A1(a. u.)	A2(a. u.)	A3(a. u.)	A4(a. u.)	τ_{enh}(ps)	τ_{r1}(ps)	τ_{fast}(ps)	τ_{r2}(ps)	τ_{slow}(ps)	τ_{r3}(ps)	τ_{r4}(ps)
10	0.088 ± 0.001	-0.150 ± 0.009	0	0.200 ± 0.010	2.10 ± 0.46	209.1 ± 4.1	0.296 ± 0.032	12.64 ± 0.69	-	-	0.08
30	0.083 ± 0.002	-0.162 ± 0.009	0	0.127 ± 0.016	1.24 ± 0.37	233.1 ± 7.1	0.280 ± 0.041	10.94 ± 0.66	-	-	0.08
50	0.072 ± 0.001	-0.127 ± 0.006	0	0.111 ± 0.011	1.03 ± 0.27	337.4 ± 8.5	0.361 ± 0.047	9.39 ± 0.47	-	-	0.08
70	0.046 ± 0.001	-0.108 ± 0.006	0	0.065 ± 0.008	1.67 ± 0.49	453.9 ± 15.9	0.244 ± 0.028	10.99 ± 0.52	-	-	0.08
90	0.020 ± 0.001	-0.064 ± 0.007	0	0.062 ± 0.012	1.88 ± 0.61	621.8 ± 22.8	0.191 ± 0.065	13.14 ± 1.39	-	-	0.08
110	0	-0.043 ± 0.009	-0.062 ± 0.002	0.098 ± 0.010	-	-	0.561 ± 0.239	1.46 ± 0.50	7.16 ± 0.63	55.2 ± 1.8	0.08
130	0	-0.029 ± 0.019	-0.166 ± 0.002	0.112 ± 0.016	-	-	0.402 ± 0.190	1.24 ± 0.42	20.99 ± 0.55	254.8 ± 5.3	0.08
150	0	0	-0.221 ± 0.001	0.092 ± 0.014	-	-	-	-	22.45 ± 0.33	6604 ± 1283	0.08
170	0	0	-0.043 ± 0.009	0.018 ± 0.011	-	-	-	-	23.92 ± 1.39	25510 ± 5055	0.08

Supplementary References

1 Dalla Longa, F., Kohlhepp, J., de Jonge, W. & Koopmans, B. Influence of photon angular momentum on ultrafast demagnetization in nickel. *Phys. Rev. B* **75**, 224431, doi:10.1103/PhysRevB.75.224431 (2007).

2 Lu, X. *et al.* Roles of heating and helicity in ultrafast all-optical magnetization switching in TbFeCo. *Appl. Phys. Lett.* **113**, 032405, doi:10.1063/1.5036720 (2018).

3 Liu, E. *et al.* Giant anomalous Hall effect in a ferromagnetic Kagome-lattice semimetal. *Nat. Phys.* **14**, 1125-1131, doi:10.1038/s41567-018-0234-5 (2018).

4 Oppeneer, P. M. & Liebsch, A. Ultrafast demagnetization in Ni: theory of magneto-optics for non-equilibrium electron distributions. *J. Phys.: Condens. Matter* **16**, 5519-5530, doi:10.1088/0953-8984/16/30/013 (2004).

5 Kirilyuk, A., Kimel, A. V. & Rasing, T. Ultrafast optical manipulation of magnetic order. *Rev. Mod. Phys.* **82**, 2731-2784, doi:10.1103/RevModPhys.82.2731 (2010).

6 Bigot, J. Y., Guidoni, L., Beaurepaire, E. & Saeta, P. N. Femtosecond Spectrotemporal Magneto-optics. *Phys. Rev. Lett.* **93**, 077401, doi:10.1103/PhysRevLett.93.077401 (2004).

7 Okamura, Y. *et al.* Giant magneto-optical responses in magnetic Weyl semimetal Co3Sn2S2. *Nat. Commun.* **11**, 4619, doi:10.1038/s41467-020-18470-0 (2020).

8 Tengdin, P. *et al.* Critical behavior within 20 fs drives the out-of-equilibrium laser-induced magnetic phase transition in nickel. *Sci. Adv.* **4**, eaap9744, doi:10.1126/sciadv.aap9744 (2018).

9 You, W. *et al.* Revealing the Nature of the Ultrafast Magnetic Phase Transition in Ni by Correlating Extreme Ultraviolet Magneto-Optic and Photoemission Spectroscopies. *Phys. Rev. Lett.* **121**, 077204, doi:10.1103/PhysRevLett.121.077204 (2018).

10 Wang, J. *et al.* Ultrafast enhancement of ferromagnetism via photoexcited holes in GaMnAs. *Phys. Rev. Lett.* **98**, 217401, doi:10.1103/PhysRevLett.98.217401 (2007).

11 Matsubara, M. *et al.* Ultrafast photoinduced insulator-ferromagnet transition in the perovskite manganite Gd0.55Sr0.45MnO3. *Phys. Rev. Lett.* **99**, 207401, doi:10.1103/PhysRevLett.99.207401 (2007).

12 Zhang, Q. *et al.* Unusual Exchange Couplings and Intermediate Temperature Weyl State in Co3Sn2S2. *Phys. Rev. Lett.* **127**, 117201, doi:10.1103/PhysRevLett.127.117201 (2021).

13 Rudolf, D. *et al.* Ultrafast magnetization enhancement in metallic multilayers driven by superdiffusive spin current. *Nat. Commun.* **3**, 1037, doi:10.1038/ncomms2029 (2012).

14 Yang, R. *et al.* Magnetization-Induced Band Shift in Ferromagnetic Weyl Semimetal Co3Sn2S2. *Phys. Rev. Lett.* **124**, 077403, doi:10.1103/PhysRevLett.124.077403 (2020).

15 Sun, F. *et al.* Spin-polarized gap in the magnetic Weyl semimetal Co3Sn2S2. *Phys. Rev. B* **104**, L100301, doi:10.1103/PhysRevB.104.L100301 (2021).

16 Zhang, Q., Nurmikko, A. V., Miao, G. X., Xiao, G. & Gupta, A. Ultrafast spin-dynamics in half-metallicCrO2thin films. *Phys. Rev. B* **74**, 064414, doi:10.1103/PhysRevB.74.064414 (2006).

17 Ogasawara, T. *et al.* General Features of Photoinduced Spin Dynamics in Ferromagnetic and Ferrimagnetic Compounds. *Phys. Rev. Lett.* **94**, 087202, doi:10.1103/PhysRevLett.94.087202 (2005).

18 Mann, A. *et al.* Insights into Ultrafast Demagnetization in Pseudogap Half-Metals. *Phys. Rev. X* **2**, 041008, doi:10.1103/PhysRevX.2.041008 (2012).

19 Muller, G. M. *et al.* Spin polarization in half-metals probed by femtosecond spin excitation. *Nat. Mater.* **8**, 56-61, doi:10.1038/nmat2341 (2009).
20 Hubner, W. & Bennemann, K. H. Simple theory for spin-lattice relaxation in metallic rare-earth ferromagnets. *Phys. Rev. B* **53**, 3422-3427 (1996).
